# Striatal phosphodiesterase 10A and medial prefrontal cortical thickness in patients with schizophrenia: a PET and MRI study

**DOI:** 10.1038/tp.2017.11

**Published:** 2017-03-07

**Authors:** R Bodén, J Persson, A Wall, M Lubberink, L Ekselius, E-M Larsson, G Antoni

**Affiliations:** 1 Department of Neuroscience, Psychiatry, Uppsala University, Uppsala, Sweden; 2 Department of Surgical Sciences, Radiology, Uppsala University, Uppsala, Sweden; 3 Department of Medicinal Chemistry, Uppsala University, Uppsala, Sweden

## Abstract

The enzyme phosphodiesterase 10A (PDE10A) is abundant in striatal medium spiny neurons and has been implicated in the pathophysiology of schizophrenia in animal models and is investigated as a possible new pharmacological treatment target. A reduction of prefrontal cortical thickness is common in schizophrenia, but how this relates to PDE10A expression is unknown. Our study aim was to compare, we believe for the first time, the striatal non-displaceable binding potential (BP_ND_) of the new validated PDE10A ligand [^11^C]Lu AE92686 between patients with schizophrenia and healthy controls. Furthermore, we aimed to assess the correlation of PDE10A BP_ND_ to cortical thickness. Sixteen healthy male controls and 10 male patients with schizophrenia treated with clozapine, olanzapine or quetiapine were investigated with positron emission tomography (PET) and magnetic resonance imaging (MRI). Striatal binding potential (BP_ND_) of [^11^C]Lu AE92686 was acquired through dynamic PET scans and cortical thickness by structural MRI. Clinical assessments of symptoms and cognitive function were performed and the antipsychotic dosage was recorded. Patients with schizophrenia had a significantly lower BP_ND_ of [^11^C]Lu AE92686 in striatum (*P*=0.003) than healthy controls. The striatal BP_ND_ significantly correlated to cortical thickness in the medial prefrontal cortex and superior frontal gyrus across patients with schizophrenia and healthy controls. No significant correlation was observed between the BP_ND_ for [^11^C]Lu AE92686 in striatum and age, schizophrenia symptoms, antipsychotic dosage, coffee consumption, smoking, duration of illness or cognitive function in the patients. In conclusion, PDE10A may be important for functioning in the striato-cortical interaction and in the pathophysiology of schizophrenia.

## Introduction

Schizophrenia is a severe mental illness with persisting or recurring symptoms and considerable disability.^1^ The underlying neurobiology of schizophrenia is not fully understood, but the dopaminergic system has been implicated. Schizophrenia symptoms comprise overactivity of the dopamine 2 (D_2_) receptor system, associated with positive psychotic symptoms such as delusions and hallucinations, as well as underactivity of the dopamine 1 (D_1_) receptor system, related to the so-called negative symptoms such as anhedonia, apathy and affective flattening.^1^ However, all available pharmacological schizophrenia treatments act mainly through blocking dopamine D_2_ receptors, which may induce parkinsonism that could mimic negative symptoms.

The striatum is a central hub in the brain networks implicated in the wide range of schizophrenia symptoms.^2, 3, 4, 5^ In striatum, the enzyme phosphodiesterase 10A (PDE10A) is found in high concentrations within the medium spiny neurons, and it is crucial for the degradation of cyclic AMP and cyclic GMP.^6^ Medium spiny neurons are the principal striatal neurons, either expressing D_1_ receptors in the direct signaling pathway or D_2_ receptors in the indirect pathway.^7^ PDE10A has opposing effects on the output from these striatal pathways by regulating second messenger cascades, and inhibition of PDE10A does indeed seem to reduce positive and in some studies also negative symptoms in animal models of schizophrenia.^8, 9, 10, 11, 12^ Moreover, patients with other striatal disorders, such as Parkinson's and Huntington's disease, have lower availability of striatal PDE10A early in the disease course.^13, 14^

Although PDE10A inhibition has shown promise as a novel target of the treatment of schizophrenia,^15^ recently the first positron emission tomography (PET) study of PDE10A expression in patients with schizophrenia found no difference compared to healthy controls using the [^11^C]IMA107 ligand.^16^ However, the sample size was small and did not include treatment-resistant patients treated with diazepines such as clozapine. Thus, it might be too early to dismiss PDE10A as a key factor in schizophrenia pathophysiology or PDE10A inhibition as a potential new treatment. Other PDE10A ligands are available and a recently validated ligand for human use with high sensitivity is [^11^C]Lu AE92686,^17^ but it has never before been used in patients with schizophrenia.

Altered function of the striatum is central to the underlying pathophysiology of schizophrenia and consequently many treatments target this region. Nevertheless, widespread cortical thinning is frequently observed and already present early in the disease, and may be both progressive and genetically driven.^18, 19^ Thus, schizophrenia is best characterized neurologically at a network level. Indeed, the striatum is well interconnected with the neocortex,^7^ and widespread alterations in striatal functional connectivity related to symptom severity have been observed in schizophrenia.^4^ In rats, it was recently found that the medial prefrontal cortex regulates striatal activity by modulating dopamine release, thereby providing a link between cortical dysfunction and striatal abnormalities in schizophrenia.^20^ It is therefore possible that altered PDE10A expression would be accompanied by cortical alterations such as the commonly observed cortical thinning, though the direction of such a relationship is unclear.

The primary aim of our study was to evaluate the expression of PDE10A with the newly validated and sensitive ligand [^11^C]Lu AE92686 in patients with schizophrenia treated with diazepines compared to healthy controls. A secondary aim was to assess any relationship between striatal PDE10A expression and brain-wide neocortical thickness.

## Materials and methods

### Subjects

Ten male patients with a clinical diagnosis of schizophrenia aged 18–45 years were recruited from the outpatient psychosis clinic at Uppsala University Hospital, Sweden. It was confirmed that the patients met the Diagnostic and Statistical Manual of Mental Disorders, 4th edition criteria for a diagnosis of schizophrenia through a Mini-International Neuropsychiatric Interview.^21^ Only patients with >2 years duration of illness and on a stable antipsychotic regimen with clozapine, olanzapine or quetiapine in the past 2 months were deemed eligible. Sixteen healthy, non-smoking male controls aged 18–45 years with no history of psychiatric or neurological disorders were recruited by advertisement. This sample size was deemed sufficient for this pilot study. All participants provided written informed consent. The study was evaluated and approved by the Research Ethical Review Board in Uppsala.

### Clinical assessment

The patients were screened for addiction through the Alcohol Use Disorders Identification Test (AUDIT),^22^ the Drug Use Disorders Identification Test (DUDIT)^23^ and urine drug screening. Coffee and nicotine consumption were assessed through weekly follow-back questionnaires. Antipsychotic dosages were determined through chart review and ingested dose confirmed through interviews with the patients and monitoring of antipsychotic blood levels. The dosages were converted to olanzapine equivalents to enable comparison.^24^ Symptoms were assessed using the Brief Psychiatric Rating Scale, expanded 24 item version,^25^ and the Clinical Assessment Interview for Negative Symptoms.^26^ We chose to use the Clinical Assessment Interview for Negative Symptoms as it is a comprehensive, yet feasible scale to deliver, developed to cover several of the Research Domain Criteria from the National Institute of Mental Health. It covers all five negative symptoms domains and separates consummatory and anticipatory anhedonia. The patients also completed a brief computerized cognitive test battery including the Digit Symbol Substitution Task.^27^

### PET and MRI

The healthy controls were instructed to refrain from coffee and alcohol 48 h prior to the PET scan, but the patients were only instructed to refrain from alcohol. The PET tracer [^11^C]Lu AE92686 was synthetized as previously described.^17^ All PET investigations were performed on an ECAT EXACT HR+ scanner (Siemens, Siemens Medical Solutions, Knoxville, TN, USA). After a 10-min transmission scan with rotating ^68^Ge rod sources, 385±86 MBq of [^11^C]-LU AE92686 were administered intravenously as a bolus injection. Simultaneously, a dynamic emission scan was started in three-dimensional mode, consisting of 25 time frames with progressive frame duration (6 × 10, 3 × 20, 2 × 30, 2 × 60, 2 × 150, 4 × 300 and 6 × 600 s) and a total duration of 90 min. Images were reconstructed using normalization and attenuation-weighted ordered subsets expectation maximization (six iterations and eight subsets), applying all appropriate corrections and a 4-mm Hanning filter, into 63 slices with a 128 × 128 matrix and a pixel size of 2 × 2 × 2.4 mm.

T1-weighted anatomical images were acquired in different scanners for the control group and patient group. In the patient group, magnetic resonance imaging (MRI) was performed on a 3 T scanner (Philips Achieva, Philips Medical Systems, Best, Netherlands), with a 32-channel head coil. Images were acquired using a 3D Turbo Field Echo sequence (repetition time=8.2 ms; echo time=3.8 ms; flip angle=8° field of view=256 × 256 mm^2^; voxel size=1 mm^3^ isotropic voxels; 220 slices, axial acquisition). For controls, a 1.5 T scanner (Philips Achieva, Philips Medical Systems) with an eight-channel head coil was used. Images were acquired using a 3D Turbo Field Echo sequence (repetition time=7.1 ms; echo time=3.2 ms; flip angle=8° field of view=256 × 256 mm^2^; voxel size=1 mm^3^ isotropic voxels; 173 slices, sagittal acquisition). MRI was used to identify anatomical structures, for cortical thickness measurements and to exclude clinically significant brain abnormalities. The PET data enabled assessment of PDE10A non-displaceable binding potential (BP_ND_).

### PET and MR data processing

Frame-by-frame realignment of the PET scans to correct for movements of the subject's head during scanning was performed using VOIager 2.0.5 (GE Healthcare, GE Healthcare Life Sciences, Little Chalfont, UK, 2009). The T1-weighted MRI images were segmented and co-registered to the PET images using SPM8. A gray matter volume of interest (VOI) for cerebellum was defined on the MRI images using an automated probabilistic atlas (PVElab) and projected over all frames of the dynamic PET scans, resulting in a time–activity curve for cerebellar gray matter.^28^ Parametric images were computed using receptor parametric mapping, a basis function implementation of the simplified reference tissue model using cerebellar gray matter as reference tissue,^29, 30^ and previously validated for [^11^C]Lu AE92686.^17^ To extract individual BP_ND_ values for basal ganglia subregions and thalamus, the individual BP_ND_ volumes were non-linearly warped to Montreal Neurological Institute standard space using a unified segmentation approach with the algorithm ‘new segment' in SPM12 (http://www.fil.ion.ucl.ac.uk/spm/), running in MATLAB R2016a (http://se.mathworks.com/products/matlab/).^31^Mean BP_ND_ values were then extracted from VOI defined with the CIC atlas: globus pallidus, thalamus, substantia nigra, nucleus accumbens, caudate nucleus and putamen.^32^ The resulting BP_ND_ values VOI wise BP_ND_ estimates were corrected for partial volume effects. Group differences in BP_ND_ were assessed for each VOI, using Mann–Whitney *U-*tests and differences were considered significant at *P*_FWE_<0.05, using Holm–Bonferroni correction. A striatal VOI was then created by combining the nucleus accumbens, caudate nucleus and putamen volumes. VOIs and atlas were chosen based on a recent publication, allowing direct comparison of findings.^16^ Whole-brain BP_ND_ maps for the entire sample were entered into a one-sample *t*-test, after smoothing with a Gaussian kernel of 8 mm full width at half maximum, for a graphic representation of brain regions where PDE10 expression was significantly above zero.

To relate striatal BP_ND_ to whole-brain cortical thickness, Freesurfer 5.3.0 (http://surfer.nmr.mgh.harvard.edu/) was used to generate cortical thickness maps. The procedures have been detailed previously.^33, 34^ In brief, the preprocessing involves stripping of non-brain tissue and subcortical structures, reconstruction of the boundary between white and gray matter and the cortical surface, and registration to a spherical atlas after inflation based on individual cortical folding patterns, allowing accurate comparison of cortical thickness across the mantle. For statistical analysis, the Query, Design, Estimate, Contrast (QDEC) application was used to fit a general linear model to each vertex, with group and striatal BP_ND_ as regressors. Vertices showing a significant relationship between striatal BP_ND_ and cortical thickness, after taking group/scanner type into account, were identified using a vertex-wise threshold of *P*_FDR_<0.05 and a cluster threshold of *P*<0.01 to correct for multiple comparisons.

### Main outcome measures

BP_ND_ of [^11^C]Lu AE92686 in striatum and its subregions.

### Statistical analyses

To assess group differences in BP_ND_, Mann–Whitney *U-*tests were calculated for each VOI. To further account for potential confounding factors, mean striatal BP_ND_ in the patient group was defined by averaging over globus pallidus, nucleus accumbens, caudate nucleus and putamen. Spearman rank correlations were then calculated to assess the relationship between striatal BP and duration of illness, medication, age and coffee consumption on the day of scanning. Striatal BP was also compared between smoking and non-smoking patients with a Mann–Whitney *U-*test. For all analyses above, a *P*-value of <0.05 was considered significant. Statistical analyses were performed in R version 3.2.2 (https://www.R-project.org/).

## Results

### Descriptives

The characteristics of the 10 patients and the 16 healthy comparison subjects are summarized in Table 1. The patients were older, consumed more coffee and smoked more.

### Main results PDE10A

**Figure 1:**
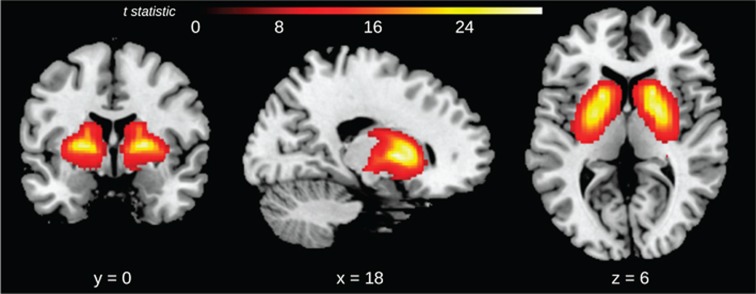
Brain regions where [^11^C]Lu AE92686 binding potential significantly differs from zero (*P*_FWE_<0.05) for the whole sample (*N*=26) as assessed with one-sample *t*-tests. Clusters include the entire striatum and globus pallidus bilaterally.

[^11^C]Lu AE92686 binding potential differed significantly from zero in striatum bilaterally when considering the whole sample including patients and controls, using a one-sample *t*-test, *P*_FWE_<0.05 (Figure 1).

**Figure 2:**
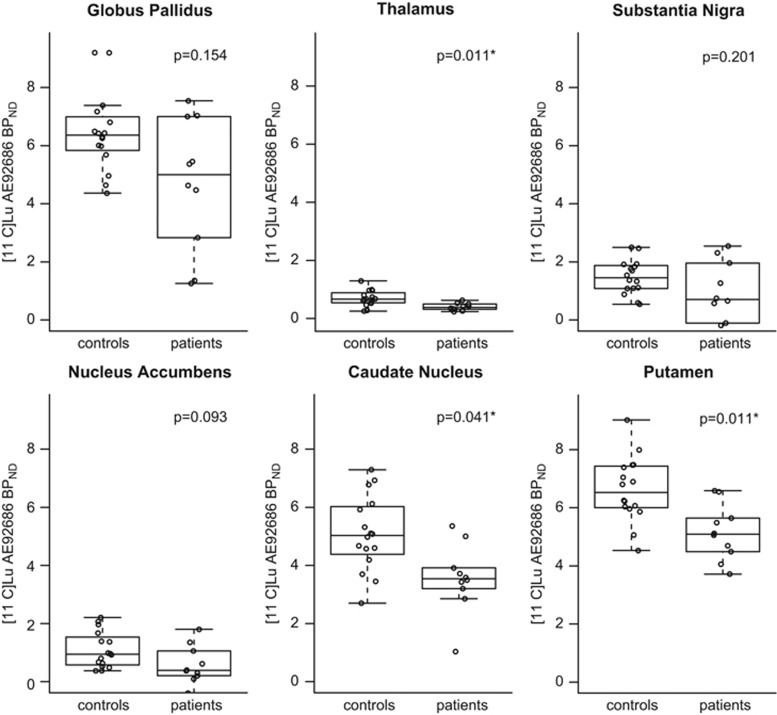
Binding potential (BP_ND_) of the phosphodiesterase 10A ligand [^11^C]Lu AE92686 in 10 patients with schizophrenia and 16 healthy controls in different basal ganglia regions and thalamus.

Mean striatal BP_ND_ of [^11^C]Lu AE92686 was significantly lower in patients compared to the healthy comparison group (*P*=0.003). Figure 2 depicts further analyses of the subregions of the basal ganglia and thalamus. Significantly lower BP_ND_ of [^11^C]Lu AE92686 was observed in the striatum, including caudate nucleus and putamen in patients compared to controls (all *P*<0.01). No significant difference was observed in globus pallidus (*P*=0.053) and substantia nigra (*P*=0.201), but here as well, the median levels were numerically lower in patients compared to controls.

**Figure 3:**
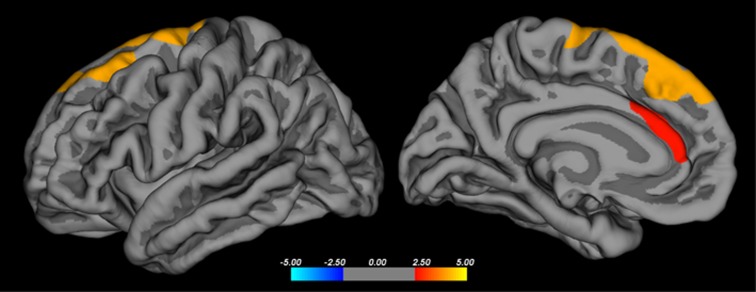
Brain regions where cortical thickness shows a positive correlation with striatal [^11^C]Lu AE92686 binding potential in the whole sample (*n*=26) after controlling for group/scanner. A vertex-wise threshold of *P*_FDR_<0.05 and a cluster threshold of *P*<0.01 was used to correct for multiple comparisons. The clusters cover the left superior frontal gyrus and the anterior cingulate cortex. FDR, false discovery rate.

**Figure 4:**
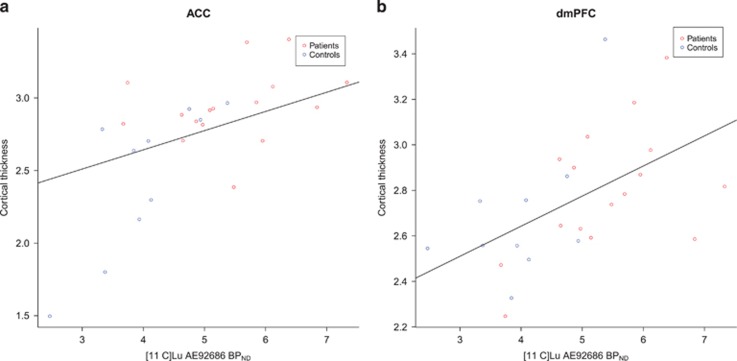
The relationship between cortical thickness and striatal [^11^C]Lu AE92686 binding potential at the point of maximal correlation within the anterior cingulate cortex (ACC) (**a**) and dorsomedial prefrontal cortex (dmPFC) (**b**), for illustrative purposes only. Individual values are color-coded according to group and fit line is based on the entire sample.

A positive correlation between cortical thickness and striatal BP_ND_ of [^11^C]Lu AE92686 in the whole sample (*n*=26) was observed in the left superior frontal gyrus, including the dorsomedial prefrontal cortex, as well as in the anterior cingulate cortex after controlling for group/scanner and age (Figures 3 and 4).

### Correlations of clinical variables and PDE10A availability

No significant correlation between age and mean striatal BP_ND_ of [^11^C]Lu AE92686 was observed in the patient group (*ρ*=−0.09, *P*=0.80). However, in the control group, increased age was associated with decreased striatal BP_ND_ (*ρ*=−0.52, *P*=0.04). Furthermore, in the patient group, no significant correlation was observed between present coffee consumption, duration of illness, olanzapine equivalents, symptoms (total Brief Psychiatric Rating Scale or Clinical Assessment Interview for Negative Symptoms scores), or digit symbol substitution task performance and BP_ND_ of [^11^C]Lu AE92686 (all *P*-values>0.05). Smokers and non-smokers did not differ in striatal BP_ND_.

## Discussion

### Key result

For we believe the first time, we show that patients with schizophrenia treated with diazepines have a significantly lower BP_ND_ of [^11^C]Lu AE92686 in the striatum compared to healthy control subjects, indicating a lower availability of PDE10A. We have also shown that higher striatal BP_ND_ is associated with greater cortical thickness in the medial prefrontal cortex and superior frontal gyrus across both groups. This finding highlights the role of PDE10A in striato-cortical interactions and suggests that functional striatal alterations and cortical thinning are part of a common underlying pathophysiology in schizophrenia.

### Strengths and limitations

We have used a sensitive and selective radiotracer for PDE10A that has been validated in rodents, primates and humans. A limitation of this study is that we only included males, which precludes the generalizability of the results to female patients with schizophrenia. Furthermore, patients and healthy control subjects differed in age. However, no significant correlation was observed between BP_ND_ of [^11^C]Lu AE92686 and age in the patient group. Similarly, neither previous studies of patients with schizophrenia (RN Gunn, personal communication)^16^ or Parkinson's disease, nor experimental animal models have established a correlation between age and PDE10A availability.^13, 35^ Another limitation was the difference in coffee and smoking habits between the two groups. This was a trade-off, as we aimed to capture a clinically relevant sample of patients with chronic schizophrenia and many of them use cigarettes and coffee. However, to our knowledge, no significant pharmacological effect of nicotine or caffeine on PDE10A availability has been reported. To speculate, smoking would, through dopamine release, probably result in an increase of PDE10, as shown by d-amphetamine challenge, by Ooms *et al.*^36^ As the patient group includes smokers and the control group does not, the effect would then probably be a reduction of the difference between the two groups due to the potential dopamine release-induced increase of PDE10 in the former group. This applies to caffeine as well, which also through its antagonist action on A2a receptors likely would increase striatal dopamine levels.^37^ Further, only the patient group was assessed with the cognitive test battery, which precluded between-group comparisons.

### Interpretation

Changes in PDE10 expression could either be part of the underlying pathophysiology of schizophrenia or possibly an effect of chronic antipsychotic treatment. Altering PDE10A expression through pharmacological inhibition or gene knockout procedures affects psychosis-like behaviors in animal models of schizophrenia.^8, 12, 38^ Thus, altered PDE10A expression would be expected as part of the underlying pathophysiology, with which our findings are consistent. However, PDE10A inhibition has been proposed as a potential treatment, given its ability to alleviate psychotic symptoms and increase social interaction in rodent models of schizophrenia.^8, 11, 39^ At first glance, this is difficult to reconcile with our findings of a lower availability of PDE10A in patients. However, PDE10A inhibition has also been associated with decreased appetitive or aversive conditioning in rodents,^38^ and decreased attention, interest and cognitive function in primates.^40^ Thus, PDE10A inhibition may actually induce negative symptoms in some circumstances. One possibility is that both over- and under-expression of this enzyme are associated with schizophrenia symptoms and yet another reason may be that animal model findings are difficult to generalize to patients with schizophrenia. It is also possible that antipsychotic pharmacological treatment underlies the apparent lower BP_ND_ of PDE10A in patients, through direct or indirect inhibition of PDE10A, in which case the observed difference compared to controls reflects a treatment effect. Continuing along this line, most of the patients in our study were treatment-resistant and therefore treated with clozapine; the others were treated with other diazepines with a similar, but not as marked balance between striatal D_1_ and D_2_ receptor occupancy.^41^ This might explain why we observed a lower BP_ND_ of PDE10A in patients with schizophrenia, whereas Marques *et al.*^16^ did not, as they studied patients treated with other, more D_2_-receptor-antagonistic antipsychotics. However, previous studies of the effect of antipsychotics on PDE10A availability conflict with one study reporting an increase of PDE10A in rats exposed to both haloperidol and clozapine,^42^ whereas Natesan *et al.* observed no difference in PDE10A availability after chronic haloperidol administration to rats.^10^ The latter concurs with a recent non-human primate study that did not detect any significant changes from haloperidol treatment.^43^ It is not fully clear if clozapine and the other diazepines (olanzapine and quetiapine) that the patients were treated with in our study are D_1_ agonists or antagonists, but a reasonable amount of data is pointing towards clozapine being a D_1_ agonist as reviewed by Ahlenius *et al.*^44^ PDE10 expression has recently been shown to partly be regulated by cyclic AMP levels in striatum.^36^ As it is more likely that the diazepines would activate D_1_ receptors than antagonize them and therefore lead to an increase in cyclic AMP levels, the end result should be a PDE10 increase. Nevertheless, it could potentially be that there is no measurable effect on PDE10 by D_1_ activation, as it may be balanced by the antagonism on the D_2_ receptor or that the diazepines actually mask some of the down regulation of PDE10 found in patients with schizophrenia in this study. What could be the root cause for decreased PDE10 expression in patients with schizophrenia is, however, not known. We believe that the current knowledge of PDE10 and effects of chronic treatment with diazepines suggest that there is no change or possibly an increase in PDE10 and therefore the results we present with decreased PDE10 are unlikely to be not an effect of treatment. Yet, further studies are needed to clarify antipsychotic treatment effects on PDE10 expression.

Another possible explanation for the discrepancy between the study by Marques *et al.* and ours might be found in the treatment-resistant status of the patients and not in the actual treatment. Patients with treatment-resistant schizophrenia do not have an increased level of dopamine synthesis capacity, as the patients do whose symptoms respond to conventional D_2_ blocking antipsychotic treatments.^45^ We did not observe any correlation between PDE10A expression and symptom severity in patients. This is congruent with the observation on the effect of PDE10A inhibition in non-human primates, which was all or none in nature.^40^ Thus, individual differences in PDE10A availability may not necessarily reflect the severity of schizophrenia, but symptoms may rather be present below a certain level of PDE10A availability. Another reason for the lack of a relationship may again be the effect of antipsychotics, whereby they block the aversive effect of low availability of PDE10A without restoring PDE10A levels. Other explanations for the discrepant findings are that we only investigated males and had an older and thus probably more chronic patient sample; PDE10A alterations might be more pronounced later in the course of the illness. [^11^C]Lu AE92686 also shows higher brain signals than the radiotracer [^11^C]IMA107 used by Marques *et al.*, and excellent reproducibility, and thus might be more sensitive to detecting group differences.^17, 46^

In other striatal disorders, such as Parkinson's and Huntington's disease, similarly lower availability of striatal PDE10A has been reported before any volumetric signs of degeneration are obvious.^13, 14^

We explored the correlations between striatal PDE10A expression and cortical thickness to investigate the effects of a possibly altered striatal function on distant brain areas. Striatal PDE10A expression was related to superior frontal gyrus and medial frontal cortical thickness. Thinning of these brain areas has been reported early in the schizophrenia illness progression and has been hypothesized to be part of the early pathophysiological process.^18, 47^ But this could also be a result of later dynamic cerebral reorganization in patients with schizophrenia.^48^ The correlation to frontal cortical thickness highlights the importance of PDE10A in cortico-striatal interactions, suggesting that striatal functional alterations and frontal cortical thinning are part of the same underlying pathophysiology. Although cognitive deficits observed in schizophrenia are traditionally ascribed to prefrontal hypofunction, evidence suggests that associative loops connecting the prefrontal cortex and striatum are crucial for executive functions and working memory.^49^ Both striatal hyperdopaminergia and prefrontal volume reduction are observed prodromally and may be important etiological factors.^18, 50^ In mice with overexpression of striatal D_2_ receptors, cognitive deficits similar to schizophrenia symptoms and hypodopaminergia prefrontally are observed.^51^ This suggests a primary deficit in the striatum underlying prefrontal dysfunction and associated cognitive deficits in schizophrenia. Our findings are in line with this and suggest that functional alterations in the striatum as reflected by a decrease in PDE10A availability may contribute to the observed cortical structural alterations. However, whether PDE10A availability is primary or secondary to cortical thinning requires further study in the early stages of the psychotic disorder or, preferably, in a longitudinal investigation. No correlation between cortical thickness and BP was observed within the patient group separately, leaving open the possibility that the correlation observed across the whole sample is merely due to group differences in binding potential and cortical thickness. However, no group difference in cortical thickness within the superior frontal gyrus was observed, and Figure 4 reveals a large degree of overlap between groups. Second, the relationship is observed after statistically controlling for group differences. This also takes into account differences in magnetic resonance acquisition between groups, which would otherwise be a confounding factor.

In conclusion, for the first time, we have observed a lower striatal PDE10A expression in patients with schizophrenia. Additional longitudinal studies of patients with first-episode psychosis, preferably untreated, as well as first-degree relatives, are warranted to elucidate whether this is a core pathophysiological phenomenon, a rebound response to some other pathophysiology, a biological vulnerability or an effect of antipsychotic treatment of patients in early disease phase.

## Figures and Tables

**Table 1:** Characteristics of patients with schizophrenia and healthy controls at the time of the PET scan

	*Schizophrenia*	*Controls*
Number of subjects	10	16
Male, *n*	10	16
Age, years, median (IQR)	39.5 (4.25)	24 (6.25)^a^
Coffee consumption, cups, day before scan, median (IQR)	2.5 (2.75)	0
Coffee consumption, cups, same day before scan, median (IQR)	1.5 (1)	0
Current smokers, *n*	4	0
Duration of illness, years, median (IQR)	16.5 (6.25)	NA
Treatment resistant, clozapine treated, *n*^b^	7	NA
Brief Psychiatric Rating Scale score, median (IQR)	49 (8.75)	NA
Antipsychotic dosage, mg per day olanzapine equivalents, median (IQR)	13.75 (13.21)	NA
Radioactivity injected, MBq, mean (s.d.)	414 (92)	367 (79)
Molar activity, GBq μmol^−1^, mean (s.d.)	32.9 (11.9)	54.4 (33.3)

Abbreviations: IQR, interquartile range; NA, not applicable; PET, positron emission tomography.

a The difference between groups was significant at *P*<0.001 as revealed with a Mann–Whitney *U-*test (Levene's test revealed no significant difference in variance, *F*<1).

b Of the remaining three patients who were all responders, two used olanzapine and one quetiapine. Among the patients treated with clozapine, one patient had concomitant treatment with perphenazine, three with aripiprazole, one with quetiapine and one with risperidone.
